# Genome Sequence of *Psychrobacter cibarius* Strain W1

**DOI:** 10.1128/genomeA.00078-16

**Published:** 2016-05-26

**Authors:** Prem K. Raghupathi, Jakob Herschend, Henriette L. Røder, Søren J. Sørensen, Mette Burmølle

**Affiliations:** Department of Biology, Section for Microbiology, University of Copenhagen, Copenhagen, Denmark

## Abstract

Here, we report the draft genome sequence of *Psychrobacter cibarius* strain W1, which was isolated at a slaughterhouse in Denmark. The 3.63-Mb genome sequence was assembled into 241 contigs.

## GENOME ANNOUNCEMENT

*Psychrobacter cibarius* belongs to the family *Moraxellaceae* within the class of gammaproteobacteria (1). It is a Gram-negative, nonmotile, rod-shaped or coccoid, psychrophilic, and halotolerant bacterium (2). *P. cibarius* was originally isolated from fermented seafood (2). However, other species within the genus *Psychrobacter* have been identified from diverse environments (1, 3–7). Here, we present the draft assembly of *P. cibarius* strain W1, isolated from a slaughterhouse in Denmark (8). Sequencing libraries were prepared using the Nextera XT kit (Illumina, USA), according to the manufacturer’s recommendations, followed by flow cell sequencing with 2 × 250-bp paired-end reads using Illumina MiSeq (Illumina) technology. The reads were cleaned and trimmed with the CLC Genomics Workbench version 7 (CLC bio, Denmark). Both merged and unmerged reads were assembled with SPAdes 3.5.0. The assembled contigs were annotated using the RAST server (9) and RNAmmer server version 1.2 (10) to screen for rRNA genes.

The final genome assembly has an approximate coverage of 117× with a total size of 3,629,624 bp composed of 241 contigs with an average G+C content of 43.7%. Annotation of the genome predicted 3,097 coding sequences (CDSs) (1,437 CDSs have functional predictions), 52 RNA genes, 2 copies of 5S and 16S rRNA genes, and one copy of the 23S rRNA gene. The closest neighbor identified by functional comparison on the RAST server was *P. cryohalolentis* K5 (score, 540) strain. There are 405 subsystems represented in the genome, and metabolic reconstruction revealed genes involved in central carbohydrate metabolism, genes for stress responses including oxidative stress and cold and heat shock. The analysis additionally identified genes responsible for resistance to bacteriocins, resistance to antibiotics and toxic compounds, copper homeostasis, copper tolerance, cobalt-zinc-cadmium resistance, resistance to fluoroquinolones, arsenic resistance, and multidrug resistance efflux pump subsystems. The existence of these genes indicates the adaptation of strain *P. cibarius* W1 to environments with high anthropogenic impact, and future work with the genomes of *Psychrobacter* spp. will give more insights on the evolution and adaptation of these bacteria to diverse environments.

### Nucleotide sequence accession numbers.

The whole-genome shotgun projects for *P. cibarius* strain W1 have been deposited at the European Nucleotide Archive (ENA) under the contig accession numbers CZJV02000001 to CZJV02000241. The versions described in this paper are the first versions.

## References

[1] Juni E, Heym GA 1986 *Psychrobacter immobilis* gen. nov., sp. nov.: genospecies composed of gram-negative, aerobic, oxidase-positive coccobacilli. Int J Syst Bacteriol 36:388–391. doi:10.1099/00207713-36-3-388.

[2] Jung S-Y, Lee MH, Oh TK, Park YH, Yoon JH 2005 *Psychrobacter cibarius* sp. nov., isolated from jeotgal, a traditional Korean fermented seafood. Int J Syst Evol Microbiol 55:577–582. doi:10.1099/ijs.0.63398-0. 15774627

[3] Shaw BG, Latty JB 1988 A numerical taxonomic study of non-motile non-fermentative Gram-negative bacteria from foods. J Appl Bacteriol 65:7–21. doi:10.1111/j.1365-2672.1988.tb04311.x. 3209519

[4] Romanenko LA, Schumann P, Rohde M, Lysenko AM, Mikhailov VV, Stackebrandt E 2002 *Psychrobacter submarinus* sp. nov. and *Psychrobacter marincola* sp. nov., psychrophilic halophiles from marine environments. Int J Syst Evol Microbiol 52:1291–1297. doi:10.1099/00207713-52-4-1291. 12148642

[5] Shivaji S, Reddy GS, Suresh K, Gupta P, Chintalapati S, Schumann P, Stackebrandt E, Matsumoto GI 2005 *Psychrobacter vallis* sp. nov. and *Psychrobacter aquaticus* sp. nov., from Antarctica. Int J Syst Evol Microbiol 55:757–762. doi:10.1099/ijs.0.03030-0. 15774658

[6] Bowman JP, Nichols DS, McMeekin TA 1997 *Psychrobacter glacincola* sp. nov., a halotolerant, psychrophilic bacterium isolated from Antarctic sea ice. Syst Appl Microbiol 20:209–215. doi:10.1016/S0723-2020(97)80067-7.

[7] Bakermans C, Ayala-del-Río HL, Ponder MA, Vishnivetskaya T, Gilichinsky D, Thomashow MF, Tiedje JM 2006 *Psychrobacter cryohalolentis* sp. nov. and *Psychrobacter arcticus* sp. nov., isolated from Siberian permafrost. Int J Syst Evol Microbiol 56:1285–1291. doi:10.1099/ijs.0.64043-0. 16738105

[8] Røder HL, Raghupathi PK, Herschend J, Brejnrod A, Knøchel S, Sørensen SJ, Burmølle M 2015 Interspecies interactions result in enhanced biofilm formation by co-cultures of bacteria isolated from a food processing environment. Food Microbiol 51:18–24. doi:10.1016/j.fm.2015.04.008. 26187823

[9] Overbeek R, Olson R, Pusch GD, Olsen GJ, Davis JJ, Disz T, Edwards RA, Gerdes S, Parrello B, Shukla M, Vonstein V, Wattam AR, Xia F, Stevens R 2014 The SEED and the Rapid Annotation of microbial genomes using Subsystems Technology (RAST). Nucleic Acids Res 42:D206–D214. doi:10.1093/nar/gkt1226. 24293654 PMC3965101

[10] Lagesen K, Hallin P, Rødland EA, Staerfeldt H-H, Rognes T, Ussery DW 2007 RNAmmer: consistent and rapid annotation of ribosomal RNA genes. Nucleic Acids Res 35:3100–3108. doi:10.1093/nar/gkm160. 17452365 PMC1888812

